# Biomonitoring in the Era of the Exposome

**DOI:** 10.1289/EHP474

**Published:** 2016-07-06

**Authors:** Kristine K. Dennis, Elizabeth Marder, David M. Balshaw, Yuxia Cui, Michael A. Lynes, Gary J. Patti, Stephen M. Rappaport, Daniel T. Shaughnessy, Martine Vrijheid, Dana Boyd Barr

**Affiliations:** 1 Department of Environmental Health, Rollins School of Public Health, Emory University, Atlanta, Georgia, USA; 2 Exposure, Response, and Technology Branch, Division of Extramural Research and Training, National Institute of Environmental Health Sciences, National Institutes of Health, Department of Health and Human Services, Research Triangle Park, North Carolina, USA; 3 Department of Molecular and Cell Biology, College of Liberal Arts and Sciences, University of Connecticut, Storrs, Connecticut, USA; 4 Department of Chemistry, and; 5 Department of Medicine, Washington University, St. Louis, Missouri, USA; 6 Department of Environmental Health Sciences, School of Public Health, University of California, Berkeley, Berkeley, California, USA; 7 Centre for Research in Environmental Epidemiology (CREAL), Barcelona, Spain

## Abstract

**Background:**

The term “exposome” was coined in 2005 to underscore the importance of the environment to human health and to bring research efforts in line with those on the human genome. The ability to characterize environmental exposures through biomonitoring is key to exposome research efforts.

**Objectives:**

Our objectives were to describe why traditional and nontraditional (exposomic) biomonitoring are both critical in studies aiming to capture the exposome and to make recommendations on how to transition exposure research toward exposomic approaches. We describe the biomonitoring needs of exposome research and approaches and recommendations that will help fill the gaps in the current science.

**Discussion:**

Traditional and exposomic biomonitoring approaches have key advantages and disadvantages for assessing exposure. Exposomic approaches differ from traditional biomonitoring methods in that they can include all exposures of potential health significance, whether from endogenous or exogenous sources. Issues of sample availability and quality, identification of unknown analytes, capture of nonpersistent chemicals, integration of methods, and statistical assessment of increasingly complex data sets remain challenges that must continue to be addressed.

**Conclusions:**

To understand the complexity of exposures faced throughout the lifespan, both traditional and nontraditional biomonitoring methods should be used. Through hybrid approaches and the integration of emerging techniques, biomonitoring strategies can be maximized in research to define the exposome.

**Citation:**

Dennis KK, Marder E, Balshaw DM, Cui Y, Lynes MA, Patti GJ, Rappaport SM, Shaughnessy DT, Vrijheid M, Barr DB. 2017. Biomonitoring in the era of the exposome. Environ Health Perspect 125:502–510; http://dx.doi.org/10.1289/EHP474

## Introduction

More than ten years ago, shortly after the human genome was sequenced, Christopher Wild proposed an environmental complement to the genome in determining risk of disease, termed the exposome. He defined the exposome as the totality of exposures throughout the lifespan (Wild 2005).

Since the exposome was originally defined, research efforts have begun, leading to a revised working definition that may be summarized by the following elements. The exposome includes the cumulative measure of exposures to both chemical and nonchemical agents such as diet, stress, and sociobehavioral factors. It includes a series of quantitative and repeated metrics of exposures—both endogenous and exogenous—that describe, holistically, environmental influences or exposure over a lifetime (from conception to death). The exposome can include traditional measures of exposure (e.g., traditional biomonitoring, environmental monitoring) but also includes untargeted discovery of unknown chemicals of biological importance (Miller and Jones 2014; Rappaport and Smith 2010; Wild 2005, 2012). Exposomic approaches go a step beyond traditional biomonitoring, aiming to capture all exposures that potentially affect health and disease.

As a cancer epidemiologist, Wild understood the importance of the environment to health and that current disease trends cannot be explained by genetics alone (Wild 2005). We are only beginning to understand the complexities of environmental exposures and their impacts on human health, whereas genetic influences on health have been extensively studied. At present, we have limited estimates of the impact of environmental exposures on health, and uncertainty exists even in those (Jones 2016; Rappaport 2016; Rappaport and Smith 2010). Biomonitoring serves as a key tool to define exposure–disease risks given the biological significance of internal exposure measurements. With the continued advancement of methods, biomonitoring strategies will be critical in achieving a comprehensive understanding of exposures that have personal and public health relevance. With full understanding of the complex interactions between genetics and environmental exposures, the mysteries of the etiology, trends, and prevention of many diseases can be solved.

In an effort to advance the framework for developing exposome approaches and characterization, a diverse group of scientists gathered at the National Institute of Environmental Health Sciences (NIEHS) Exposome Workshop in January 2015 to discuss the current state of the science and to provide recommendations to the environmental health sciences community on how to best advance exposome research. The state of the science along with the perspectives and recommendations of our working group, Biomonitoring for the Exposome, are described here.

## Discussion

### Traditional Biomonitoring

Exposure is commonly assessed by a spectrum of questionnaire data and ecological, environmental, or biological measurements. Biological measures of exposure that determine an internalized dose are often preferred because they are usually more relevant to the health outcome studied. Traditional biological measurements, also called targeted analyses, measure a target chemical, metabolite, or reaction product in a biological medium such as urine or blood (see Appendix 1). These traditional biomonitoring measurements have become a key component of exposure assessment in many epidemiologic studies that attempt to link exposures to health outcomes.

**Appendix 1:** Glossary.

Term	Definition
Traditional biomonitoring/targeted analyses	Analyses of biological samples for specific chemicals: either exposures or markers of exposures
Semi-targeted/hybrid approaches	Exploits the advantages of both targeted and untargeted approaches: for example, using metabolomics for discovery of potential exposures followed by targeted analysis for a more fully quantitative measure
Multiplexing	Fractionation of samples to remove higher-level chemicals, enabling detection of lower-abundance chemicals
Untargeted analyses	Agnostic analyses that can measure a broad set of endogenous and exogenous metabolites in one sample run
Feature	A raw data output from mass spectrometry analysis which includes an accurate mass-to-charge ratio with associated retention time (RT) and ion intensity; a feature can represent one or more chemicals/metabolites, so data extraction methods are critical to interpretation
Biomonitoring	Can refer to measurement of chemicals through both targeted and untargeted methods
High-resolution metabolomics	A mass spectrometry technique that can detect >10,000 features through instrumentation such as time-of-flight, Fourier transform ion cyclotron resonance and orbitrap mass analyzers
HELIX (The Human Early-Life Exposome)	A European-funded project under the Seventh Framework Programme for Research and Technological Development (FP7) Exposome Programme focused on understanding the early-life exposome through novel exposure measurement and data-driven methods
HERCULES (Health and Exposome Research Center: Understanding Lifetime Exposures)	A National Institute of Environmental Health Sciences–funded center at Emory University focused on providing infrastructure and expertise to develop and refine new tools and technologies to advance exposome research as well as on promoting environmental health sciences research overall
EXPOsOMICS	A European-funded project under the FP7 Exposome Programme that aims to develop a new approach to assessing environmental exposures in adults, particularly through the use of omics techniques
HEALS (Health and Environment-wide Associations based on Large population Surveys)	A European Commission–funded project focused on integrating omics data and traditional biomonitoring measurements with alterations in outcomes such as gene expression and metabolic regulation to assess environmental exposures and human health associations

Molecular epidemiology studies and regulatory agencies rely primarily on targeted analyses because of their current availability and historical use. Broad surveys such as the National Health and Nutrition Examination Study (NHANES) utilize these methods, allowing for quantification and longitudinal surveillance of known exposures across the U.S. population. NHANES data facilitate comparative identification of abnormal exposure levels in select population subsets. Major epidemiology studies such as those evaluating blood lead levels and mean IQ in children and prenatal pesticide exposures and neurological deficits in children and neurodegenerative disease in adults have linked significant health outcomes to specific exposures, informing opportunities for further mechanistic studies (Chin-Chan et al. 2015; Kaufman et al. 2014; Rosas and Eskenazi 2008). Other federal efforts in the United States include the National Biomonitoring Program (NBP) of the Division of Laboratory Sciences at the Centers for Disease Control and Prevention (CDC). The NBP produces a National Report on Human Exposure to Environmental Chemicals and regularly updates the NHANES biomonitoring data in that report (CDC 2009, 2015). Chemicals of potential concern such as arsenic, perchlorate, and environmental phenols, among others, continue to be added to NHANES, with the most recent report including data on > 250 chemicals. The CDC also provides grant funding to a variety of state laboratories to increase public health laboratory capacity for surveillance. Targeted analytical capabilities and worldwide use continue to expand through both public health and academic entities.

***Historical use of biomonitoring.*** Traditional biomonitoring methods are well established for exposure assessment in epidemiology studies and in federal and state surveillance activities. Because of their historical use, they provide a number of strong advantages for exposure research (see Appendix 2). Biologically persistent chemicals are well-characterized with traditional methods, whereas short-lived chemicals are effectively measured only if the individual is undergoing continuous or continual exposures or if the timing of exposures is known. Chemicals such as phthalates, bisphenols, and parabens are well-characterized by targeted methods given their widespread use and presence in the environment. Often, chemicals of particular toxicological interest may be difficult to measure owing to barriers such as stability or presence in readily accessible biological matrices such as blood or urine. For example, short-lived chemicals such as various current-use pesticide and phthalate metabolites can only be detected in urine samples if exposure occurs within a few days of testing; therefore, continuous or longitudinal sample collection is necessary to capture exposure. For a selected group of 250–300 known persistent (~30–40%) and nonpersistent (~60–70%) chemicals, sample analysis provides exposure information for the chemical of concern within a specific window of exposure; reference data are available for most of these chemicals (CDC 2015).

**Appendix 2:** Key advantages and disadvantages of traditional biomonitoring for determination of exposure.

Traditional biomonitoring for determination of exposure	
Advantages	Disadvantages
Well-established and reliable methods for both long-lived (biologically persistent) chemicals and short-lived chemicals with continuous exposures Highly selective methods Provides accurate and precise measurements of biologically persistent chemicals Often targets known chemicals of toxicologic importance Reference data exist for most chemicals Targeted approach allows specific hypotheses of well-documented chemicals to be studied	Limited to a select group of known chemicals (~ 250) Studies such as NHANES do not take continuous measures, thereby limiting detection of short-lived chemicals Suspected chemicals of concern are less likely to be captured Time-intensive methods development and validation Chemicals added for monitoring not always the most important from a toxicologic perspective Analyses are expensive and time-consuming Few laboratories with expanded capabilities Multiple methods required for a large suite of chemicals Typically requires 500–2,000 μL of blood or other biospecimens for each chemical analyzed

The ~250 chemicals that are commonly measured in the United States are primarily driven by the CDC biomonitoring list of target analytes (CDC 2015). Most other programs also follow the CDC list because selection of these agents was informed by a public nomination process followed by expert ranking of the nominated chemicals (CDC 2012). An important caveat of this process is the target list is partially based on ease of performance and compatibility with existing methods. Another concern is that some of the chemicals have little toxicological relevance or diminishing exposure across the population resulting from successful regulation of their release into the environment, or a combination of the two.

***Biomonitoring methods.*** Although method development for traditional biomonitoring can be quite rigorous, this also translates into a slow and expensive process when developing analysis protocols for new chemicals of interest. These analyses often require relatively high volumes of sample, typically 0.5–1 mL for a single method (~ 10 mL urine and > 20 mL serum to measure the 250–300 currently biomonitored chemicals), which can be limiting for certain biospecimen types and age groups under study. For exposome research, these requirements restrict the number and types of chemicals that can be measured at any one time. Unknown or suspected chemicals of concern may not be measured or identifiable through targeted methods (see Appendix 2) (Rappaport et al. 2014); yet targeted analyses are valuable given the accuracy and depth at which a chemical of interest can be assessed. By coupling traditional biomonitoring methods with broader exposomic approaches, the benefits of both strategies can be fully realized.

### Exposomic Approaches

An exposomic approach differs from traditional biomonitoring in that it can theoretically include all exposures of potential health significance, whether they are derived from exogenous sources (e.g., pollutants, diet, drugs) or endogenous sources (e.g., hormones, human and microbial metabolites) (Rappaport and Smith 2010; Rappaport et al. 2014). Because levels of chemicals in blood or other biospecimens reflect a wide range of exposures or the metabolic consequences of exposures, including psychosocial stress, other nonchemical stressors such as noise, and nutritional factors, exposomic biomonitoring offers an efficient means for characterizing individual exposure profiles. Incorporating the exposome paradigm into traditional biomonitoring approaches offers a means to improve exposure assessment in many ways (Wild 2012).

***Untargeted analyses.*** With only a few hundred chemicals routinely assessable through targeted methods and with limitations for short-lived compounds, exposomic approaches are critical to understanding the thousands of chemicals people are exposed to daily through direct chemical exposures or consequences of exposure (e.g., cortisol levels due to stress or noise exposures) (CDC 2015). Through untargeted biomonitoring approaches such as high-resolution metabolomics (HRM), > 1,500 metabolites can be monitored with a relatively small amount of biological specimen (≤ 100 μL) and for the cost of a single traditional biomonitoring analysis of 8–10 target chemicals (Johnson et al. 2010; Jones 2016).

Untargeted analyses of small molecules or macromolecular adducts in blood, urine, or other matrices are well suited for exposome-wide association studies (EWAS), which compare profiles of hundreds or thousands of chemical features—analogous to ions with a given mass-to-charge ratio and a specified retention time in traditional biomonitoring —between diseased and healthy subjects (Rappaport 2012, 2016). Indeed, untargeted analyses performed using the current generation of liquid chromatography–high resolution mass spectrometers (LC-HRMSs) can detect > 30,000 small-molecule features (Ivanisevic et al. 2013) and > 100 human serum albumin (HSA) adducts of reactive electrophilic chemicals (including reactive oxygen species) at the nucleophilic locus Cys34 (Grigoryan et al. 2012; Rappaport et al. 2012). Processing the rich sets of data from untargeted analyses of archived biospecimens offers a path for discovering health-impairing exposures that have thus far escaped scrutiny, a largely unrecognized benefit of exposomics. It is important to note that full annotation of molecular features is not required for case–control comparisons provided that LC-HRMS signatures are available (e.g., accurate mass, retention time, and MS/MS fragmentation). Archived biospecimens from well-designed cohort studies already exist. With continued advancement in untargeted analyses, there is potential to make significant advances in human health through uncovering unknown exposures (da Silva et al. 2015; Zhou et al. 2012).

***High-resolution metabolomics.*** Although untargeted analyses encompass a wide range of the -omics techniques, HRM is a technique that is poised to advance exposomics research because of the breadth of coverage it offers of both endogenous and exogenous chemicals. At the present time, it is routine to detect tens of thousands of features with HRM, and this number will increase as the sensitivity of mass analyzers continues to improve. These features do not necessarily represent different chemical constituents but provide extensive data for evaluation of alterations in biological pathways (Mahieu et al. 2014). Extensive comparisons of the features of these various instruments are available elsewhere (Marshall and Hendrickson 2008). With the additional advancements that have been made in bioinformatics methods to aid in feature extraction and data analysis, HRM has become an increasingly viable tool for broad exposome-level characterization (Jones 2016). Although features linked to human health will require chemical identification, the technology is in place for the feature extraction methods and annotation efforts that will increase the total number of chemicals that can be monitored (Soltow et al. 2013). Researchers are already demonstrating this expanded potential along with the ability to quantify chemicals under a high-resolution metabolomics platform (Go et al. 2015; Li et al. 2015). By definition, untargeted approaches are agnostic, allowing unknown or emerging exposures of concern (see Appendix 3) to be detected. These approaches are often hypothesis-generating and may require testing of newly discovered analytes/exposures in experimental models to confirm effects on biological responses.

**Appendix 3:** Key advantages and disadvantages of exposomic approaches for determination of exposure.

Exposomic approaches for determination of exposure	
Advantages	Disadvantages
Agnostic approaches are encouraged for detection of emerging exposures of concern Techniques (and development of techniques) promote identification of unknown/emerging exposures of concern Links exogenous exposures to internal biochemical perturbations A large number of features can be detected (> 10,000) for the cost of a single traditional biomonitoring analysis Includes biomolecular reaction products (e.g., protein adducts, DNA adducts) for which traditional biomonitoring measurements are often lacking or cumbersome Requires a small amount of biologic specimen (~ 100 μL or less) for full-suite analysis Enables detection of “features” that are linked to exposure or disease for further confirmation Encourages techniques to capture short-lived chemicals Aims to measure biologically meaningful lifetime exposures, both exogenous and endogenous, of health relevance	Agnostic approach can be problematic for grant funding May not detect chemicals present at low levels Cannot detect all analytes present in chemical space A reference or baseline value may not be possible to define Extensive bioinformatics required for data reduction/analysis Requires carefully collected and well-maintained biospecimens Can only measure chemicals that are isolated in extraction process (e.g., acetonitrile extraction would not necessarily capture lipophilic chemicals) Relies heavily upon library searching of spectra for annotation with standard confirmation coming later, which can be quite time-consuming and labor-intensive May be difficult to link measures to exposure source Includes lifetime exposures but does not place enough emphasis on defining and measuring windows of susceptibility (e.g., *in utero*) to accurately capture the most biologically important exposures

***Detection of low-level xenobiotic exposures.*** Persistent challenges exist with detecting chemicals present at low levels, defining reference values of “normal” exposure, and ultimately linking these measures to an exogenous source so intervention can occur. Because blood concentrations of xenobiotics (femtomolar to micromolar) tend to be much lower than those of chemicals derived from food, drugs, and endogenous sources (nanomolar to micromolar), untargeted analyses of xenobiotics are not as efficient and reliable as those of ingested and endogenous sources at detecting many exposures of interest (Rappaport et al. 2014). To determine the health impacts of these exposures, it will be necessary to develop semi-targeted or multiplexed methods that increase the signals of exogenous molecules relative to those of endogenous origin (Rappaport et al. 2014; Southam et al. 2014; Wei et al. 2010). Analyses of suspected chemicals of concern, also referred to as suspect screening, can be prioritized through measuring panels of chemicals with known biological effects but without identifying a specific hypothesis regarding the toxicological pathway. Untargeted and suspected chemical analyses both fall under exposomic biomonitoring and offer extraordinary potential for increased understanding of complex chemical exposures.

***Hybrid approaches.*** Various terms are used to describe hybrid approaches, including suspect screening or semi-targeted analyses. Because both targeted and untargeted approaches have beneficial attributes as well as drawbacks, using a hybrid exposomics approach may enable us to exploit advantages while minimizing the limitations of each technique. One of the obvious limitations of a targeted approach is its inability to provide exposure information on a wide array of chemicals. However, targeted analysis can typically provide validated and quality-assured detection and quantification at very low concentrations that may not be available using an untargeted approach until HRM and the necessary bioinformatic data extraction techniques mature. As mentioned above, the development of these quantitative techniques for HRM is underway with advancements in instrumentation (Go et al. 2015; Marshall and Hendrickson 2008). Furthermore, the generic extraction methods used in untargeted analysis may not be able to capture all of the chemicals of interest (e.g., limited extraction of nonpolar chemicals using a typically polar solvent extraction), whereas more specialized extractions can specifically target chemical classes.

***Semi-targeted analysis.*** Semi-targeted analysis can utilize various approaches including a two-step strategy: discovery using metabolomics followed by a more fully quantitative targeted measure. Another potential approach would involve a known or measured chemical exposure in individuals for which metabolomic measurements could also be made. For instance, untargeted metabolomic analysis of each group would then allow for a search for new exposure biomarkers and unique metabolic pathway pertubations to help elucidate the effect mechanism.

Traditionally, targeted analysis data have been used for risk assessment purposes, so shifting solely to a newer platform may take some time. The hybrid approach can be useful for both exposomic analysis and informing targeted analysis approaches. For example, a targeted chemical concentration can be used as an “outcome” for metabolome-wide association studies (e.g., evaluating biochemical alterations relative to targeted chemical concentrations), or a metabolomic analysis can help identify important chemicals that need to be rigorously quantified for health or risk assessments. Of course, each of the two approaches stands on its own, and they have done so for many decades. By combining the two, however, we have a much more powerful approach to understanding chemical exposures, biological alterations, and disease.

### Overarching Issues

***Matrix selection.*** Whether using a traditional biomonitoring or an exposomic approach, careful attention must be given to which matrices can be practically collected and which matrices are relevant for assessing chemical exposures. The matrices available for collection during different life stages and a nonexhaustive list of the chemicals that are appreciably present in these matrices have been reviewed elsewhere (Barr et al. 2005). Typically, the least-invasive matrix in which the chemicals appreciably collect, such as blood and urine, is the preferred matrix.

Although most analysis of exposure is performed with urine or blood samples as a consequence of the ease with which they can be collected, there are other sample types that have begun to be explored for their value in exposome interrogation. For example, saliva, which can be collected from school-age children and adults, is a problematic matrix to collect from infants and toddlers because of choking dangers associated with the collection devices and the inability of young children to actively secrete it. Even if the matrix, in this case saliva, can be noninvasively collected, the target chemical or suite of potential chemicals may not enter the matrix for a variety of reasons, including protein binding of chemicals that prevents their secretion into saliva (Lu et al. 1998). In addition, saliva is nonsterile; therefore, contributions of the oral microbiome can influence the composition of the analytes to be measured. Buccal and nasal swabs have also been used to assess the biological consequences of external exposures. In those sample types, DNA, mRNA, and their adducts have been the principal focus to date (Beane et al. 2011; Spira et al. 2004; Zhang et al. 2010), but these samples (as well as fecal samples) are also compromised by the presence of a strong microbial community that can influence the composition of the exposome constituents.

Other biological samples (e.g., selected blood cells, sweat, teeth, nails) can include information about recent historical exposures in their composition. The use of alternative samples as historical measures of exposure may become important in future studies. Teeth are a matrix that has demonstrated particular promise for characterizing prenatal exposures to metals and to some organic chemicals because of their defined growth patterns (Andra et al. 2015). We can use the “record” of prior exposures recorded in hair, deciduous teeth, or molecular “fingerprints” in other samples to provide historical measures of certain exposures (Arora et al. 2012; Hu et al. 2007); however, validation of the time represented in the exposure history may be laborious.

There are limitations to these sample sets because external deposits of specific chemicals can make the interpretation of measured levels in these samples different from those measured in blood, for example. In addition, standardized protocols and reference standards are lacking for many alternative matrices, making standardization of results across studies difficult.

An important consideration when choosing samples for exposome-type research is the anticipated presence of the particular chemical(s) in the harvested samples. Because chemicals may display unanticipated pharmacodynamics and biotransformation, it may ultimately be essential that multiple sample types are collected from each individual in the effort to define the exposome. Blood circulates throughout the body, so there is an advantage to its assessment because it has been exposed to the myriad of routes by which an environmental chemical may enter the body. However, some analytes are known to specifically accumulate in particular tissues; thus, a broad-spectrum assessment of multiple patient samples will provide the best insights into exposures.

***Analytical considerations for matrix effects.*** In addition to the relevant matrices that can be collected, we must consider the alterations in response that may be obtained in analytic systems related to other components of the matrix. Such matrix effects can enhance analytic signals or work to suppress signals (Panuwet et al. 2016). In fact, each individual sample will exert its own matrix effects that can make quantification difficult, particularly in mass spectrometry–based methods. Mass spectrometers are inherently sensitive to matrix effects such that the analytical signal of a given concentration can vary over orders of magnitude if appropriate internal standards for normalizing the mass spectral signal have not been used (Baker et al. 2005). In particular, these variations could present challenges when attempting to quantify features in untargeted analysis approaches.

***Sample collection and storage.*** Collection and storage procedures are particularly important considerations for internal exposure measurements. Failures in the proper collection and storage of specimens can result in lost sample integrity, samples that are not suitable for analysis, and contamination/degradation of important chemicals. Because of the sensitivity of some methods such as HRM, biospecimens must be carefully collected and well-maintained. Specific attention to freeze-thaw cycles, potential contamination risks, and collection protocols is needed to ensure that the data extracted from each sample are accurate. It is nearly impossible to control for every preanalytic challenge in sample collection and storage for an untargeted analysis, which is one reason that both targeted and untargeted analyses are quite complementary. In addition, both targeted and untargeted approaches can only measure a limited amount of the exogenous and endogenous chemicals that are present in our bodies. The types and number of chemicals within us that are measureable largely depend upon the matrix selected and the method used [Children’s Health Exposure Analysis Resource (CHEAR) 2016a, 2016b].

***Variability of exposures.* Temporal variability.** Temporal, spatial, and genetic variability and variability in biological distribution of chemicals are important elements to characterize in internal exposure studies. It is important to understand if a single sample obtained at a given life stage represents average exposure over time [e.g., a blood sample for dichlorodiphenyldichloroethylene (DDE) measurements during adulthood obtained during a time of much physiologic change, such as pregnancy], or if peak exposures during a critical window are more important to consider. For short-lived chemicals, new technologies and approaches that facilitate collection of real-time data, high-dimensional analyses, and uncovering biological response markers of transient exposures offer strategies for capturing historically difficult measurements (Dennis et al. 2016).

**Spatial variability.** In addition, it is important to understand how temporal variability may vary over geographic areas and in different exposure scenarios. In this respect, exposure assessment can become very complex. Multiple samples within a population are generally preferred over a single sample so that both temporal and spatial variability can be assessed; however, the collection of multiple samples is often cost-prohibitive and can be an undue burden on participants. To appropriately interpret internal exposure data in the context of risk or health outcome, it is imperative to ascertain the degree of variability in space and time.

**Pharmacodynamic variability.** Ideally, we would have information on variability in pharmacodynamics to potentially evaluate resulting exposure data (e.g., whether a given chemical distributes to tissues differently among individuals). Most of the pharmacodynamic information we have for specific chemicals is derived from animal studies, and these processes may not be the same in humans. In addition to exposure and pharmacokinetic variability, laboratory and sampling variability should also be assessed and, if possible, teased apart from true intra-person variability.

***Fit-for-purpose use.*** The “fit-for-purpose” concept has gained popularity in traditional biomonitoring (Lee et al. 2006). This concept addresses the balance between overall cost of analysis and the degree of analytical rigor required to use the internal exposure measure results for a given purpose. In instances where legal implications exist or regulatory decisions are to be made, maximum analytical rigor is required. But for exploratory studies and for many epidemiologic studies, statistical power derived from a larger number of samples, but with sufficient precision to detect differences, is often preferred. In these cases, relaxation of analytical rigor may translate into lower costs that, in turn, could enable the number of samples analyzed to increase. Furthermore, in untargeted approaches, authentic standards are not always necessary to evaluate a chemical’s relationship to disease or alterations in biomolecular concentrations. In addition, many “add-on” studies use samples collected for different analyses for which the sample collection/storage may represent more imprecision, thus not warranting the increased cost of strict analytical rigor.

For each given study or study question, it is important to consider the analysis and the criteria that are necessary to meet the study objectives. For example, if a study seeks to control for smoking but needs validation of the questionnaire, a low-resolution method such as an immunoassay for molecular indications of smoking may be most suitable for the study; this would maximize the money available for other needs in the study. Many times, substantial resources are dedicated to perfecting an analytic method rather than using a portion of those funds to determine which measurements are actually critical to answering a research question. The issue of balance in analytic rigor and cost needs to be addressed in each study.

Extant data also represent a “fit-for-purpose” approach. Extant data were often collected to answer a certain set of research questions; thus, they are not always applicable to a different study question. However, extant data do represent a source for generating hypotheses that can be further tested using prospective, longitudinal studies. For example, NHANES data offer a resource to evaluate the extent of U.S. population exposures to particular chemicals and can serve as a tool for the exposure component of risk assessment. Although the data are cross-sectional, they serve as a useful hypothesis-generating resource.

***Unknown analytes.*** Characterizing unknown analytes remains a major challenge for understanding the exposome. Research efforts should prioritize the development of methods to determine relevant exposures and to identify sources of specific chemical signatures. By linking shifts in the microbiome, the metabolome, the proteome, and so forth to unknown analytes, we can start to determine the profiles of unknown toxicant exposures and their consequences. Additionally, biomonitoring techniques that can assess changes in cellular composition or in the developmental capacity of cells may indicate risks for later health conditions such as cancer and neurodegenerative diseases. Even if the identity of an analyte is unknown, linking unknown exposures to potential disease consequences creates further support for the investment of resources necessary to understanding cumulative lifetime exposures.

Annotation of spectra for unknown chemicals can be quite time-consuming and therefore only completed on a select number of features. Limitations regarding chemical annotation will best be overcome through a concerted effort across many research groups to identify, catalogue, and disseminate information related to newly identified small molecules. Additionally, continued focus on bioinformatics techniques to extract information about chemical features of importance will allow semi-targeted approaches to be used for unknown and low-abundance chemicals.

The omics technologies all have potential for discovering unknown analytes. Through ongoing advancements in mass spectrometry, low-abundance chemicals can be targeted and characterized. With comprehensive coverage of the metabolome, reference metabolic profiles combined with health outcome data would provide a baseline for identification of unknown analytes with health relevance. Through a concerted effort across laboratories, identification and cataloguing unknown analytes will become a tangible task for advancing the exposome.

### Overcoming Gaps and Barriers to Exposome Research

Several data gaps or barriers exist in both targeted and untargeted analyses. For untargeted analyses, the ability to identify and quantify low-abundance analytes—most environmental chemicals—is still immature. Untargeted approaches may need new, more sensitive mass spectrometric approaches or chemoselective probes to improve the detection of low-abundance chemicals. We reemphasize that analytic standards are not required for discovery of new and relevant biomarkers; they become necessary only when a new biomarker is identified and needs to be validated.

Although many biomonitoring resources are available through public health and academic laboratories, few laboratories exist with the capacity to measure a wide array of “known” toxicants, particularly in nonstandard matrices (i.e., matrices other than blood and urine) (see Appendix 4). Having access to such capacity is particularly important for new investigators, who may not have established relationships with such laboratories. Additionally, accurate and reproducible measures across laboratories remain a challenge. The CHEAR initiative, led by the National Institute of Environmental Health Sciences, represents a unique opportunity to provide a standardized laboratory network with access to targeted and untargeted analyses of biospecimens and so may serve to fill these gaps (NIEHS 2015).

**Appendix 4:** Biomonitoring resources.

Category	Resource/location	Website
Targeted	Centers for Disease Control and Prevention (CDC) National Biomonitoring Program	http://www.cdc.gov/biomonitoring/
	National Exposure Research Laboratory at U.S. Environmental Protection Agency (EPA )	http://www.epa.gov/nerl/
	LRN-C Laboratory Response Network for Chemical Threats	http://emergency.cdc.gov/lrn/chemical.asp
	Laboratory for Exposure Assessment and Development for Environmental Research (LEADER), Emory University	www.leaderlaboratory.org
	Chemical Analysis Facility Core, Rutgers University	http://eohsi.rutgers.edu/core-facilities/chemical-analysis-facility-core/
	Biomarker Mass Spectrometry Facility, University of North Carolina	http://sph.unc.edu/cehs/facility-cores/bms-sub-core/
	QB3/Chemistry Mass Spectrometry Facility, University of California, Berkeley	http://qb3.berkeley.edu/qb3/msf/
	Environmental Health Laboratory and Trace Organics Analysis Center, University of Washington	http://depts.washington.edu/ehlab/
	Clinical Pharmacology Analytical Services, University of Minnesota	https://www.pharmacy.umn.edu/research/service-labs/clinical-pharmacology-analytical-services-laboratory
	Biomarker Core, Center for Tobacco Control Research and Education, University of California, San Francisco	https://tobacco.ucsf.edu/core-c-biomarker-core
	Analytical Chemistry Core, Superfund Research Center, Duke University	http://sites.nicholas.duke.edu/superfund/cores/analytical-chemistry-core/
	Children’s Health Exposure Analysis Resource (CHEAR): National Exposure Laboratory Network	https://chearprogram.org/about/labnetwork
Untargeted	Wishart Research Group, University of Alberta	http://www.wishartlab.com/
	Berkeley Center for Exposure Biology, University of California, Berkeley	http://circle.berkeley.edu/research/exposome/
	Clinical Biomarkers Lab, Emory University	http://clinicalmetabolomics.org/
	West Coast Metabolomics Center, University of California-Davis	http://metabolomics.ucdavis.edu/
	Michigan Regional Comprehensive Metabolomics Resource Core, University of Michigan, Ann Arbor	http://mrc2.umich.edu/
	Eastern Regional Comprehensive Metabolomics Resource Core, RTI International, Research Triangle Park	https://chhe.research.ncsu.edu/facility-cores/systems-technologies-core/metabolomics/
	Southeast Center for Integrated Metabolomics, University of Florida, Gainesville	http://secim.ufl.edu/
	Resource Center for Stable Isotope-Resolved Metabolomics, University of Kentucky, Lexington	http://bioinformatics.cesb.uky.edu/bin/view/RCSIRM/
	Mayo Clinic Metabolomics Resource Core, Rochester, MN	http://www.mayo.edu/research/centers-programs/metabolomics-resource-core/overview
	CHEAR: National Exposure Laboratory Network	https://chearprogram.org/about/labnetwork
Funding/biomonitoring support	CDC funded state biomonitoring grants in 2009 and 2014 (CA, NY, WA, MA, NH, NJ, VA, UT, AZ, CO, NM)	http://www.cdc.gov/biomonitoring/state_grants.html
	Alaska State Public Health Laboratories	http://dhss.alaska.gov/dph/Labs/Pages/chemistry/default.aspx
	Rocky Mountain Biomonitoring Consortium Projects	https://www.colorado.gov/pacific/cdphe/rocky-mountain-biomonitoring-consortium-projects
	National Institute of Environmental Health Sciences (NIEHS) Children’s Environmental Health and Disease Prevention Research Centers	https://www.niehs.nih.gov/research/supported/dert/programs/prevention/
	NIEHS Superfund Program	https://www.niehs.nih.gov/research/supported/dert/programs/srp/index.cfm
	NIEHS EHS Core Centers Program	https://www.niehs.nih.gov/research/supported/dert/programs/core/index.cfm
	Association of Public Health Laboratories	https://www.aphl.org/programs/environmental_health/Pages/default.aspx
	Association of State and Territorial Health Officials	http://www.astho.org/Programs/Environmental-Health/
	American Association of Poison Control Centers	http://www.aapcc.org/about/
	Council of State and Territorial Epidemiologists	http://www.cste.org/?page=EHOHI
International biomonitoring labs and programs	Health Canada	http://www.hc-sc.gc.ca/ewh-semt/index-eng.php
	Human Biomonitoring Research Unit	https://www.lih.lu/page/departments/hbru-human-biomonitoring-research-unit-800
	DEMOCOPHES Harmonized Biomonitoring Surveys	http://www.eu-hbm.info/democophes
	Centre de Toxicologie/ Institut national de santé publique du Québec (INSPQ), Quebec, Canada	http://www.inspq.qc.ca/ctq/Default.asp?Page=1&Lg=en
	Dept. of Growth and Reproduction Rigshospitalet, Copenhagen, Denmark	http://www.reproduction.dk/
	Finnish Institute of Occupational Health Chemical Safety, Helsinki, Finland	http://www.ttl.fi/en/chemical_safety/Pages/default.aspx
	Institute for Prevention and Occupational Medicine, Bochum, Germany	http://www.ipa.ruhr-uni-bochum.de/e/
	Medizinisches Labor Bremen, Bremen, Germany	http://www.mlhb.de/
	Fondazione IRCCS Ca’ Granda Ospedale Maggiore Policlinico, Department of Occupational and Environmental Medicine, Milano, Italy	http://www.policlinico.mi.it/
	National Institute for Minamata Disease (NIMD), Kumamoto, Japan	http://www.nimd.go.jp/english/index.html
	Institut Hospital del Mar Medical Research Institute (IMIM), Barcelona, Spain	http://imim.es/en_index.html
	Centro Nacional de Sanidad Ambiental, Instituto de Salud Carlos III (ISCIII), Madrid, Spain	http://www.isciii.es/ISCIII/es/contenidos/fd-el-instituto/fd-organizacion/fd-estructura-directiva/fd-subdireccion-general-servicios-aplicados-formacion-investigacion/fd-centros-unidades/centro-nacional-sanidad-ambiental.shtml
	Scania University Hospital Lund Occupational and Environmental Medicine, Lund, Sweden	http://www.med.lu.se/english/department_of_laboratory_medicine/occupational_and_environmental_medicine/research

***Databases.*** The application of untargeted metabolomics to identify environmental exposures correlated with human health has its own unique challenges. The largest reference databases for metabolomics are the Metabolite and Tandem MS Database (METLIN) and the Human Metabolome Database (HMDB) (Tautenhahn et al. 2012; Wishart et al. 2009). To date, METLIN and HMDB have largely focused on naturally occurring metabolites. To our knowledge, the number of compounds in METLIN and HMDB that may be potentially relevant to exposure studies has not yet been carefully assessed. The number of databases available for metabolomics continues to expand and has unique utility depending on the research question. A more expansive discussion of metabolomics database resources is available (Go 2010). To facilitate large-scale exposomic studies, the field may benefit from having a database or from having database search functionalities specifically dedicated to environmental exposure chemicals. As discussed above, discovery experiments are typically most successful when a small subset of features can be targeted for structural identification. Thus, databases and repositories curating information on the human exposome would provide powerful mechanisms for prioritizing features of interest to environmental health scientists.

### Bioinformatic Approaches

Although bioinformatics were covered under the scope of the Biostatistics and Informatics Workgroup at the NIEHS Exposome Workshop, it is worthwhile to mention a few bioinformatic needs that are specific to the development of exposomic biomonitoring approaches. As highlighted throughout this article, characterizing the complexities of the exposome requires use of broad coverage techniques to link internal biochemical perturbations to external exposures. Bioinformatic requirements for these types of data analyses are substantial, yet they offer a high return on investment. Through pathway analysis and data extraction algorithms, biological pathway perturbations can provide great insight into broad disease processes. Additionally, detection of low-level xenobiotic and unknown chemicals can be greatly enhanced through bioinformatic techniques. Further development of bioinformatic tools and data storage and handling will be key to advancing our understanding of the health impact of complex exposures.

### Implementing the Exposome

External exposures and the actual body burden of said exposures can be quite variable. There is much to be learned about combining external and internal measures to maximize understanding of exposure and how to mitigate exposures that have negative health consequences. Coupling technologies and utilizing real-time monitoring tools can increase our overall understanding of exposures spatially and temporally. Exposome studies in Europe such as The Human Early-life Exposome (HELIX); Health and Environment-wide Association Studies based on Large population Surveys (HEALS); and EXPOsOMICS have started to demonstrate specific approaches for capturing this type of information [Community Research and Development Information Service (CORDIS) 2014, 2015; Vrijheid et al. 2014].

Similarly, Emory University’s NIEHS-funded Human Exposome Research Center: Understanding Lifetime Exposures (HERCULES) has developed infrastructure that has supported several environmental health studies using hybrid biomonitoring approaches (Go et al. 2014, 2015; Jones 2016; Zhang et al. 2014). HELIX also uses a hybrid approach for data collection. HELIX specifically focuses on cohorts of mother-child pairs to better understand which developmental periods may be particularly vulnerable to environmental exposures (Vrijheid et al. 2014). Along with personal external exposure monitoring strategies, traditional biomonitoring techniques have been combined with untargeted omics analyses (e.g., metabolomics, proteomics, transcriptomics, epigenomics) with a particular focus on repeat sampling to capture nonpersistent biomarkers. By performing omics–exposure and omics–health association studies, researchers aim to uncover biologically meaningful omics signatures. The HELIX design is one example of a current approach that integrates traditional and nontraditional techniques to better understand the exposome. Although HELIX offers one initial study structure for understanding the exposome, continued emphasis for exposomic approaches should be placed on developing techniques to measure nonpersistent chemicals that do not place undue burdens on study participants or significant financial constraints on the research study.

### Recommendations

The following recommendations are suggested for approaching internal exposure assessment for exposome research:

***Recommendation 1: Encourage secondary analyses of samples collected for traditional targeted chemical studies.*** High-quality samples (i.e., samples that have been collected and stored properly) from longitudinal epidemiology studies should be used for untargeted analysis and alternative measurement techniques. For this aim to be successful, it is critical that methods for sample collection and storage be standardized. Investment should be made in maintaining established cohorts and in developing protocols that optimize stabilization of samples for storage (e.g., does one analyte stabilizer actually destabilize other analytes of interest? Would adding a known xenobiotic act as a standard for normalization? Should multiple small aliquots be stored at the time of collection to facilitate different analytical needs?).

***Recommendation 2: Evaluate and use standardized measurement platforms with measurement harmonization.*** A general prototype platform or reference samples should be established under which different technologies can be tested. By establishing this platform, researchers can have a standardized way of demonstrating capacity with new approaches, which would allow efficient integration of effective methods into research protocols. One approach would be to use samples from NHANES or from a similarly well-characterized data set as a “challenge” or “quality control” set for new and emerging technologies. Moreover, development of or participation in multi-lab proficiency testing programs will ensure harmonization of data across studies.

***Recommendation 3: Use existing resources and databases to obtain information on current exposures that may be important.*** Significant efforts have been made to expand databases such as the HMDB, Kyoto Encyclopedia of Genes and Genomes (KEGG) human metabolic pathways, and METLIN databases (Kanehisa and Goto 2000; Kanehisa 2002; Smith et al. 2005; Wishart et al. 2009, 2013). Mining these well-developed resources in conjunction with new data analyses will enable a more comprehensive exposure characterization.

***Recommendation 4: Provide guidance for the use of existing databases and develop tools to allow searches across multiple databases.*** To facilitate researchers’ integrating exposomic approaches into their studies, resources regarding existing databases should be streamlined. Integration of existing databases such as the HMDB, LIPID MAPS Structure Database and METLIN or search options that can readily work across these resources would enhance their utility for exposome research (LIPID MAPS 2015; Smith et al. 2005; Wishart et al. 2009, 2013).

***Recommendation 5: Foster and facilitate discussion with people from different disciplines to discuss the reality of targeted and untargeted analytic capabilities.*** Discussions should focus around the development of semi-targeted or multiplexing strategies (Wei et al. 2010). Specific discussions should emphasize approaches for capturing short-lived chemicals while minimizing undue financial and participant burdens. Through generating discussion regarding established methods, researchers can have a structured dialogue concerning the utility of targeted, untargeted, and hybrid methods.

***Recommendation 6: Develop chemistry methods to enable the detection of low-abundance chemicals and to enable differentiation of endogenous molecules from exogenous molecules.*** Through methods such as multiplexing, interfering chemicals can be removed to allow detection of low-level environmental chemicals that are often difficult to detect because of higher-abundance endogenous chemicals from food, drugs, and normal metabolic processes (Rappaport et al. 2014). Investments in the development of semi-targeting or multiplexing strategies should be a high priority.

***Recommendation 7: Develop bioinformatics techniques to enhance detection of unknown chemicals using untargeted methods.*** With continued efforts such as ExpoCast, untargeted analysis can be combined with advanced bioinformatic techniques to help prioritize risk assessment, to determine which exposures often co-occur, and to establish markers of disease risk (Dennis et al. 2016; Johnson et al. 2015; Rager et al. 2016; Yu et al. 2013; Wambaugh et al. 2013).

***Recommendation 8: Encourage development of pharmacokinetic models.*** Through building simulated human response models, researchers would be able to incorporate kinetic and dynamic variability to inform interpretation of biomonitoring data.

## Conclusions

Measurable long-term improvements to human health are attainable through working towards a holistic understanding of environmental influences. In order to assess the exposome, traditional biomonitoring should be coupled with untargeted discovery of unknown chemicals of biological importance. It is critical to note that the advances described here, including those still in early stages of development, require commitment of scientific resources and energy to bring such approaches to fruition. Continued discussion and integration of approaches will be necessary to address the inherent complexity of the exposome. Broad characterization and understanding of internal exposures and their consequences are achievable under the exposome paradigm through combining emerging technologies and untargeted approaches with traditional biomonitoring techniques.
